# Dopamine subsystems that track internal states

**DOI:** 10.1038/s41586-022-04954-0

**Published:** 2022-07-13

**Authors:** James C. R. Grove, Lindsay A. Gray, Naymalis La Santa Medina, Nilla Sivakumar, Jamie S. Ahn, Timothy V. Corpuz, Joshua D. Berke, Anatol C. Kreitzer, Zachary A. Knight

**Affiliations:** 1 Department of Physiology, University of California, San Francisco, San Francisco, CA USA; 2 Kavli Institute for Fundamental Neuroscience, University of California, San Francisco, San Francisco, CA USA; 3 Neuroscience Graduate Program, University of California, San Francisco, San Francisco, CA USA; 4 Howard Hughes Medical Institute, San Francisco, CA USA; 5 Department of Neurology, University of California, San Francisco, San Francisco, CA USA; 6 Weill Institute for Neurosciences, University of California, San Francisco, San Francisco, CA USA; 7 Gladstone Institutes, San Francisco, CA USA

**Keywords:** Hypothalamus, Neural circuits

## Abstract

Food and water are rewarding in part because they satisfy our internal needs^1,2^. Dopaminergic neurons in the ventral tegmental area (VTA) are activated by gustatory rewards^3–5^, but how animals learn to associate these oral cues with the delayed physiological effects of ingestion is unknown. Here we show that individual dopaminergic neurons in the VTA respond to detection of nutrients or water at specific stages of ingestion. A major subset of dopaminergic neurons tracks changes in systemic hydration that occur tens of minutes after thirsty mice drink water, whereas different dopaminergic neurons respond to nutrients in the gastrointestinal tract. We show that information about fluid balance is transmitted to the VTA by a hypothalamic pathway and then re-routed to downstream circuits that track the oral, gastrointestinal and post-absorptive stages of ingestion. To investigate the function of these signals, we used a paradigm in which a fluid’s oral and post-absorptive effects can be independently manipulated and temporally separated. We show that mice rapidly learn to prefer one fluid over another based solely on its rehydrating ability and that this post-ingestive learning is prevented if dopaminergic neurons in the VTA are selectively silenced after consumption. These findings reveal that the midbrain dopamine system contains subsystems that track different modalities and stages of ingestion, on timescales from seconds to tens of minutes, and that this information is used to drive learning about the consequences of ingestion.

## Abstract (web summary)

Distinct dopaminergic neurons in the ventral tegmental area respond to physiological fluid balance and nutrient cues at specific stages of ingestion, driving learning about the physiological effects of ingestion.

## Main

Animals must decide what to eat and drink. This presents a challenge because the nutritional value of a food source may not be obvious from its external appearance and the consequences of its ingestion are not instantly apparent. Rather, animals must learn through ingestive experience the values of specific foods and fluids—whether they are worth exerting effort to obtain, and whether they should be consumed if encountered^6–8^. For example, many animals obtain most of their water from food^9–15^, and are therefore likely to learn through post-ingestive feedback which food sources are rehydrating.

Many aspects of learning about rewards involve dopaminergic (DA) neurons in the ventral tegmental area VTA^16–21^ (VTA-DA neurons). In response to the taste of food^3,4^ or water^4,5^, VTA-DA neurons release a burst of dopamine that confers value on associated cues. However, the taste of food and water are themselves cues that predict the subsequent satisfaction of an internal need (such as rehydration), and their value can change as animals learn about the post-ingestive effects of specific foods and fluids^1,8,22^. This raises the question of how internal nutrients and fluids, which are the final reinforcers of behaviour, are themselves represented in the dopamine system and used to drive learning.

## DA neurons track changes in hydration

**Fig. 1:**
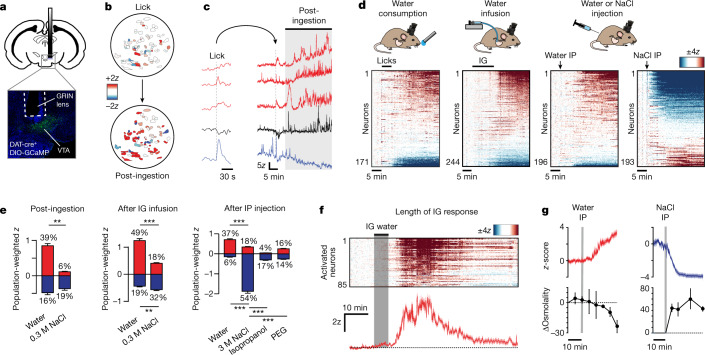
VTA-DA neurons track systemic hydration. **a**, Representative image of GRIN lens placement and VTA-DA neurons expressing GCaMP6. **b**, Tuning maps of DA neuron responses from the same field of view during and after drinking water. **c**, Example traces of calcium dynamics in five representative neurons during and after water consumption. **d**, Individual neuron responses to consumption of water, intragastric (IG) infusion of water (1.2 ml), and intraperitoneal (IP) injection of water (1.2 ml) or hypertonic saline (3 M NaCl, 0.12 ml). **e**, Population-weighted *z*-score (calculated as the fraction of neurons activated or inhibited multiplied by their *z*-scored activity change) for each of the stimuli shown. The percentages of neurons activated (red) and inhibited (blue) are listed above and below each bar graph. ‘Post-ingestion’ is from 0 to 20 min after the end of self-paced consumption of water or 0.3 M NaCl; ‘after IG infusion’ is from 0 to 20 min after the end of intragastric infusion (1.2 ml) of water or 0.3 M NaCl; ‘after IP injection’ is from 0 to 30 min after intraperitoneal injection of water (1.2 ml), NaCl (3 M, 0.12 ml), isoproterenol (100 mg kg^−1^) or polyethylene glycol (PEG) (40%, 0.4 ml). **f**, Top, time course of the activation and then return to baseline of individual VTA-DA neurons following intragastric infusion of water (1.2 ml). Bottom, mean trace. Mice are from a separate cohort than those used in **d**. **g**, Mean activity traces of neurons (from **d**) activated by intraperitoneal water injection (red) and inhibited by intraperitoneal NaCl injection (blue), with concurrent blood osmolality changes plotted below. NS, *P* > 0.05; **P < 0.01, ***P < 0.001. Data are mean ± s.e.m. Statistics are presented in Extended Data Table 2.

**Extended Data Fig. 1:**
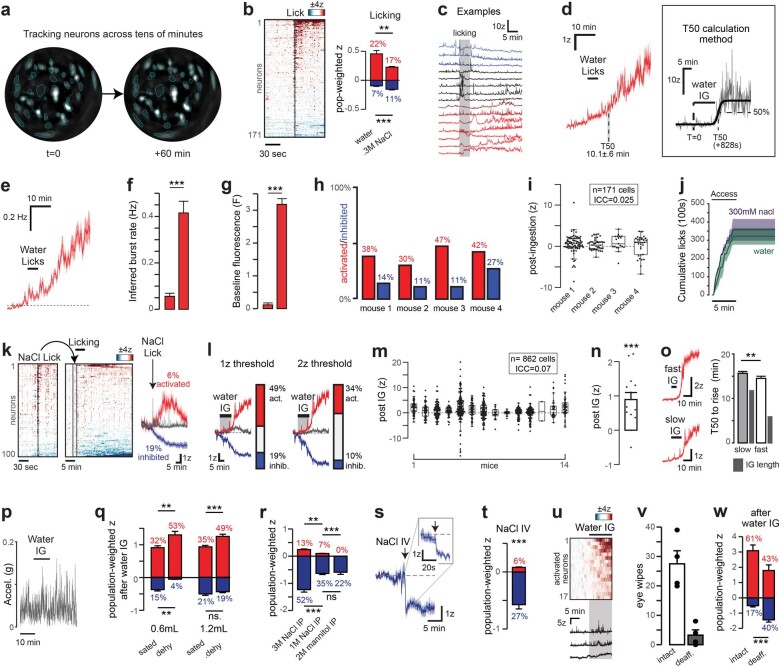
VTA-DA neurons respond to post-ingestive changes in fluid balance. **a**, Recording images separated by one hour with cell boundaries generated by CNMFe in mice not subject to manipulation. **b**, Dynamics of VTA-DA neurons during a bout of licking water and the population weighted z-score of the neurons activated (red) or inhibited (blue) from 0 to 30 s following the onset of the first licking bout. **c**, Activity traces of individual neurons during and after water consumption (see Fig. 1c for additional examples). **d**, Mean z-score of neurons activated following drinking water and illustration of how the 50% rise time (T50) is calculated for neurons responding to IG water infusion. Time of water access is shown (“water licks”). **e**, Mean inferred spike rate of DA neurons activated following water ingestion. **f,g** Inferred spike rate (**f**) and baseline fluorescence (**g**; see Methods) changes in DA neurons activated following water ingestion. **h**, Percentage of neurons activated and inhibited following water ingestion for four mice from Fig. 1d. **i**, Summary boxplots showing z-scored change of activity following water ingestion for each neuron in each of four mice (intra-class correlation or ICC=varbetween mice/vartotal). **j**, Cumulative licks for self-paced drinking of water or hypertonic saline (0.3 M NaCl) for thirsty mice (n = 4 and 3). **k**, (Left) Responses of individual neurons during and following consumption of hypertonic saline (0.3 M NaCl). (Right) Mean z-scored activity of neurons activated (red), inhibited (blue), and unaffected (grey) following saline ingestion. **l**, Mean z-scored activity during water IG infusion (from Fig. 1c) using different z-score thresholds for defining the activated and inhibited population. **m**, Summary boxplots showing z-scored change of activity following water IG infusion for each neuron separated by mouse (data comes from all recordings during water IG infusion in dehydrated mice; each replicate is a different mouse; from Fig. 1 and Extended Data Figs. 1, 6). **n**, Data points are the mean z-scored change in activity (for all neurons) for each mouse shown panel m. Bar graph shows the mean of these mean z-scores. **o**, Mean activity of neurons activated following IG infusion of water at 0.1 mL/min (slow) and 0.2 mL/min (fast) speeds and a summary plot of the T50. **p**, Mean head acceleration during and after water IG infusion (n = 3 mice). **q**, Mean responses of activated and inhibited neurons (population weighted z-score) following IG infusion of different water volumes in sated and water-deprived mice. **r**, Mean responses of activated and inhibited neurons (population weighted z-score) following IP injections of 0.12 mL of 3 M NaCl, 1 M NaCl, and 2 M mannitol. Note that 1 M NaCl and 2 M mannitol are equiosmotic. **s**, Mean response of neurons inhibited by tail vein injection of NaCl (zoom in shows a shorter timescale to illustrate the rapid response). **t**, Mean responses and proportion of neurons activated and inhibited after tail vein injection (asterisks from permutation test applied to all neurons). **u**, Dynamics of individual neurons activated during IG infusion of water (1.2 mL). **u**, Dynamics of individual neurons activated during IG infusion of water (1.2 mL). **v**, Eye wipes elicited within 15s of 0.1% capsaicin ocular delivery. This was performed three days after IP injection of 50 mg/kg capsaicin or control saline in order to functionally validate capsaicin deafferentation. **w**, Mean responses and proportion of neurons activated and inhibited after IG water infusion (1.2 mL) in dehydrated mice before and after vagal deafferentation with 50 mg/kg capsaicin. ns.P > 0.05, *P < 0.05, **P < 0.01, ***P < 0.001. All error bars show mean±s.e.m. Statistics in Extended Data Table 2.

To investigate how DA neurons respond to internal changes in fluid balance, we recorded their activity before and after water consumption. Mice were outfitted for microendoscopic imaging of calcium dynamics in VTA-DA neurons (defined by expression of DAT-cre; Fig. 1a), deprived of water overnight, and then given unrestricted access to water for 5 min. The mice drank voraciously (Extended Data Fig. 1j) and—as expected—many DA neurons were transiently activated in a manner time-locked to licking^4,5^ (22%; Extended Data Fig. 1b and Extended Data Tables 1 and 2). However, we also detected an unexpected second wave of DA neuron activation that emerged following a delay of 10.1 ± 0.6 min from the start of drinking (Fig. 1b–e and Extended Data Fig. 1c–i). This delayed activation recruited many DA neurons (39%), involved increases in both baseline fluorescence and phasic bursts, and was unrelated to licking (the water bottle was gone). Instead, it mirrored the reported time course for water absorption into the blood^23–26^, suggesting that systemic rehydration itself triggers delayed activation of many DA neurons.

To test whether this delayed response requires rehydration, we gave mice access to a hypertonic solution (300 mM NaCl) that naive mice will drink, but is ultimately dehydrating^27^. Consumption of this fluid caused transient DA neuron activation during drinking (Extended Data Fig. 1b,k) but did not result in a delayed increase of dopamine activity (Fig. 1e), despite similar levels of total consumption (Extended Data Fig. 1j). Of note, a small population of neurons was also inhibited, but the magnitude of this inhibition was the same following consumption of either water or hypertonic saline (Fig. 1e).

**Extended Data Fig. 2:**
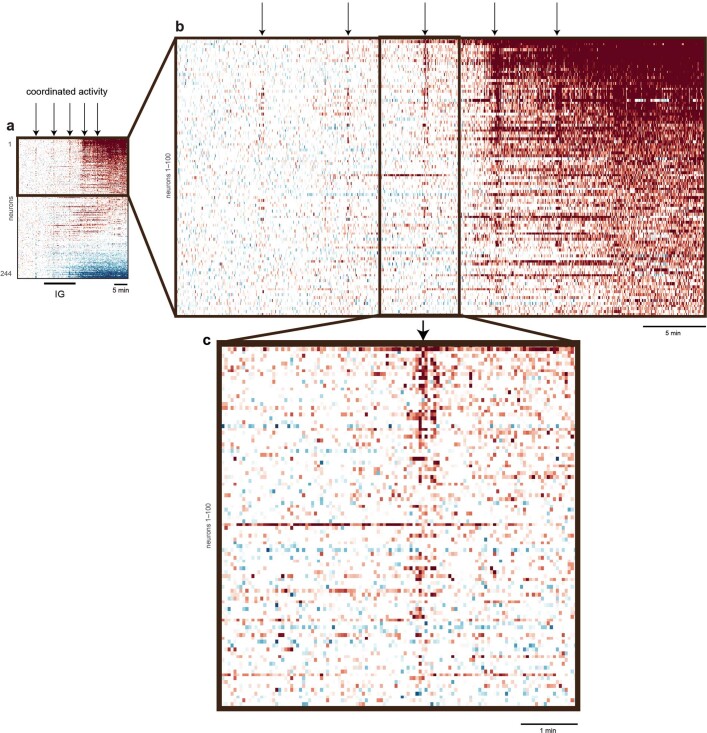
Close-up view of the activity of VTA-DA neurons during IG water infusion. **a**, VTA-DA neuron responses to IG water infusion (from Fig. 1d) exhibit instances of apparent coordinated activity when viewed on a timescale of tens of minutes. Points of apparent coordinated activity are marked with arrows. **b**, Close-up view of first 100 neurons show that not all neurons participate in this coordinated activity. **c**, Further zoomed in dynamics of the first 100 neurons show that much of this activity is uncoordinated on the seconds timescale.

To test whether rehydration is sufficient for DA neuron activation, we equipped mice with intragastric catheters for direct fluid infusion into the stomach^28^. Water infusion^29^ (1.2 ml over 12 min) resulted in delayed activation of many recorded neurons (Fig. 1d,e, Extended Data Fig. 1m,n and Extended Data Fig. 2). This occurred 14 ± 1 min after infusion onset (varying slightly with speed of infusion; Extended Data Fig. 1o) and persisted for 30 ± 0.7 min before gradually returning to baseline (Fig. 1f). The percentage of neurons activated and the magnitude of their activation were similar following self-paced drinking and intragastric infusion (Fig. 1e). Notably, this activation was not accompanied by gross changes in movement (Extended Data Fig. 1p), a smaller response was observed if the mice were not water-deprived beforehand (Extended Data Fig. 1q), and net inhibition was observed if hypertonic (300 mM NaCl) saline was infused instead (Fig. 1e), indicating that rehydration is necessary and sufficient for the DA neuron response.

Rehydration can cause changes in blood osmolality, volume and pressure, all of which influence thirst. We therefore independently manipulated these signals to determine which are responsible for DA neuron activation following water ingestion. Intraperitoneal injections of water and hypertonic saline resulted in the activation and inhibition, respectively, of many DA neurons (Fig. 1d,e), and the time course of these DA neuron responses tracked concurrent changes in blood osmolality (Fig. 1g). Injection of mannitol inhibited DA neurons similarly to equiosmotic hypertonic saline (indicating the response reflects changes in osmolality and not changes in sodium concentration; Extended Data Fig. 1r), and the DA neuron response occurred within seconds when salt was delivered intravenously (indicating that it reflects a systemic change; Extended Data Fig. 1s–t). By contrast, injections of polyethylene glycol or isoproterenol, which decrease blood volume and blood pressure, respectively^27,30,31^, caused much smaller DA neuron responses (Fig. 1e). Thus, many VTA-DA neurons specifically and bidirectionally track changes in systemic osmolality, revealing an unexpected role for fluid balance in controlling activity of the dopamine system.

## Neural responses to water and nutrients

**Extended Data Fig. 3:**
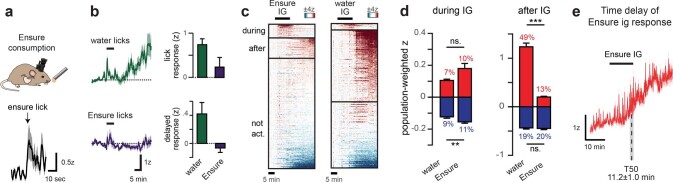
VTA-DA neuron responses to food and water occur primarily at different stages of ingestion. **a**, Mean response of VTA-DA neurons during an Ensure lick bout. **b**, Mean neuron activity during consumption of water and Ensure, depicted as the mean trace and summary plot. “Lick response” is defined as the mean z-scored change of activity from 0 to 30 s after the onset of consumption. “Delayed response” is defined as the mean z-scored change of activity from 0 to 20 min after the end of consumption. **c**, Responses of individual VTA-DA neurons during and after IG infusion of Ensure and water (1.2 mL). Neurons are separated according to whether they are activated most strongly during or after IG infusion. **d**, Mean responses of activated and inhibited neurons during and after IG infusion (population weighted z-score, as defined in Fig. 1). **e**, Rise time of neurons activated (from Extended Data Fig. 2c). ns.P > 0.05, ***P < 0.001, by permutation test. All error bars and shaded lines show mean±s.e.m.

We next compared how DA neurons respond to water and nutrients. We first confirmed that consumption of the liquid diet Ensure by hungry mice resulted in transient activation of many DA neurons time-locked to licking^3,4^ (Extended Data Fig. 3a,b). To cleanly isolate post-ingestive signals, we recorded dopamine responses to infusion of Ensure directly into the stomach (in an amount (1.2 kcal) that approximates a moderately sized meal^32^ and inhibits hunger-promoting AgRP neurons^33^). This revealed that distinct DA neuron subsets are activated during nutrient infusion (10% of DA neurons, when nutrients enter the gastrointestinal tract) and afterward (13% of DA neurons; when most nutrients are absorbed into the blood; Extended Data Fig. 3c–e). This indicates that, relative to water, nutrients activate more DA neurons while in the gastrointestinal tract, but fewer after absorption into the bloodstream (Extended Data Figs. 1u and 3c,d).

**Fig. 2:**
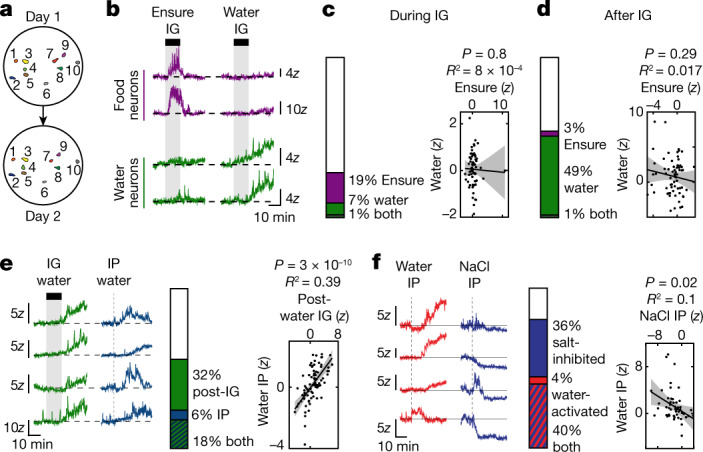
Distinct dopamine subsystems track food and water ingestion. **a**, Maps of individual VTA-DA neurons tracked across experimental days during imaging. **b**, Example traces from neurons specifically responding to intragastric infusion (1.2 ml) of either Ensure or water. **c**, Left, proportion of neurons activated during intragastric infusion of Ensure and water. Right, individual neurons show no correlation in their response during intragastric infusion of water versus Ensure. **d**, Left, proportion of neurons activated from 0–20 min after the end of intragastric infusion of water or Ensure. Right, individual neurons show no correlation in their response after intragastric infusion of water versus Ensure. **e**, Left, example traces showing neurons activated after both intragastric infusion and intraperitoneal injection of water (1.2 ml). Middle, proportion of neurons activated by intraperitoneal and intragastric water. Right, the response of individual neurons to intraperitoneal water injection is positively correlated with their response to intragastric water injection. **f**, Left, example traces showing neurons that display opposite activity patterns after intraperitoneal water injection (1.2 ml) and hypertonic saline injection (3 M NaCl, 0.12 ml). Middle, proportion of neurons activated by intraperitoneal water and inhibited by intraperitoneal saline. Right, the responses of individual neurons to intraperitoneal water and intraperitoneal saline are negatively correlated. Data are mean ± s.e.m. Statistics are shown in Extended Data Table 2.

**Extended Data Fig. 4:**
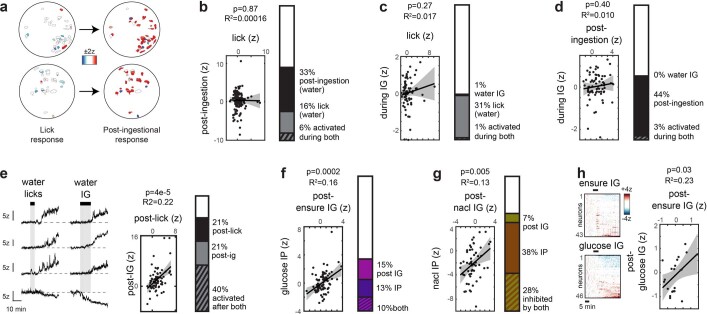
DA subpopulations respond to ingestion on three distinct timescales. **a**, Tuning maps showing individual DA neuron responses during consumption of water and post-ingestion. **b**, Proportion of neurons activated during consumption of water and post-ingestion and correlation of activity. **c**, Proportion of neurons activated during consumption of water and during IG infusion of water and correlation of activity. **d**, Proportion of neurons activated post-water ingestion and during IG infusion of water and correlation of activity. **e**, Example traces of neurons activating both after water IG infusion and after self-paced water consumption, alongside proportion of neurons activated by both and the correlation of activity. **f**, Proportion of neurons activated after Ensure IG infusion and after glucose IP injection and correlation of activity. **g**, Proportion of neurons inhibited after IG infusion and IP injection of NaCl and correlation of activity. **h**, Responses of individual neurons during IG infusion of Ensure and glucose, alongside the correlation of activity of the tracked neurons. Statistics in Extended Data Table 2.

We next compared how individual DA neurons respond to these different stimuli by aligning their responses across experiments (Fig. 2a). For drinking, individual neurons responded consistently to changes in fluid balance regardless of the method of fluid administration. For example, most neurons activated after intraperitoneal injection of water or self-paced drinking were also activated after intragastric infusion of water (Fig. 2e and Extended Data Fig. 4e). Neurons activated after intraperitoneal injection of water were correspondingly inhibited by injection of hypertonic saline (Fig. 2f). Similarly, for nutrients, the response of individual neurons after intragastric infusion of Ensure was correlated with their response after glucose injection or infusion (Extended Data Fig. 4f,h). In contrast to these consistent responses to systemic changes, there was no correlation in individual neural responses across stages of ingestion (for example, during versus after licking; Extended Data Fig. 4a–d) or between water and nutrients at the same stage (Fig. 2b–d). This reveals that there are subsets of VTA-DA neurons that are tuned to track nutrients or fluids at a specific stage of ingestion.

## Pathway linking hydration signals to VTA

**Extended Data Fig. 5:**
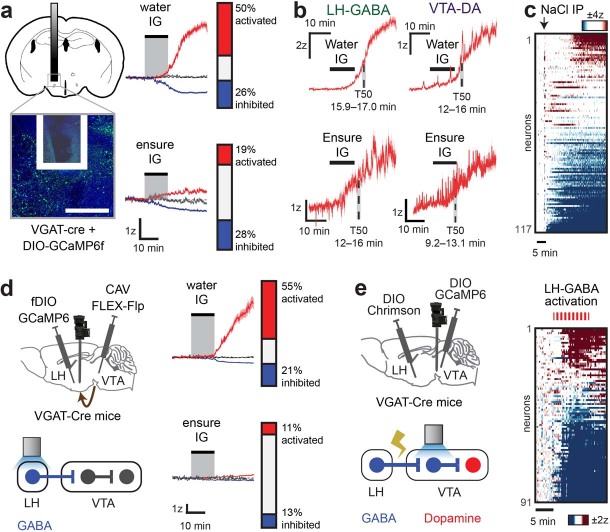
LH-GABA neurons respond to blood osmolality changes and modulate VTA dynamics. **a**, (Left) Representative image of GRIN lens placement for imaging LH neurons. Scale bar, 0.5 mm. (Right) Population-weighted z-scored activity and proportions of LH-GABA neurons activated (red), inhibited (blue), and unaffected (grey) after IG infusion (1.2 mL) of water (388 neurons/3 mice) and Ensure (169 neurons/3 mice). **b**, Rise time of LH-GABA (from a) and VTA-DA neurons (from Figs. 1e, 2b–c) activated following IG infusion of ensure or water (1.2 mL). **c**, Dynamics of individual LH-GABA neurons after IP injection of hypertonic saline (3 M NaCl, 0.12 mL; 5 mice). **d**, (Left) Schematic for microendoscope imaging of LH-GABA→VTA neurons. (Right) Split-plot and proportion of projection neurons activated, inhibited, and unaffected after infusion of water (160 neurons/3 mice) and Ensure (150 neurons/3 mice). **e**, Schematic for simultaneous microendoscope imaging of VTA-GABA neurons and optogenetic stimulation of LH-GABA neuron terminals, alongside dynamics of individual VTA-GABA neurons during LH-GABA neuron terminal stimulation (3 mice). All shaded lines show mean±s.e.m.

We next investigated how information about fluid balance is transmitted to the VTA. We first considered the lateral hypothalamus (LH), which is implicated in ingestive behaviour^34,35^ and sends a dense GABAergic (γ-aminobutyric acid-producing) projection to the VTA^36^ (that can activate VTA-DA neurons indirectly through inhibition of intervening GABAergic neurons in VTA^37^ (VTA-GABA neurons)). We prepared mice for imaging of GABAergic neurons in LH (LH-GABA neurons) and intragastric infusion and then recorded how these neurons respond to changes in fluid balance. Similar to VTA-DA neurons, many LH-GABA neurons were activated following water infusion (Extended Data Fig. 5a,b) and inhibited by injection of hypertonic saline (Extended Data Fig. 5c). To test whether the subset of LH-GABA neurons that specifically project to the VTA display these responses, we used a retrograde tracing strategy to image calcium dynamics in LH-GABA→VTA neurons, revealing that these neurons are also strongly activated following intragastric infusion of water (Extended Data Fig. 5d). Thus LH-GABA→VTA neurons track fluid balance and are poised to relay this information to the VTA.

**Fig. 3:**
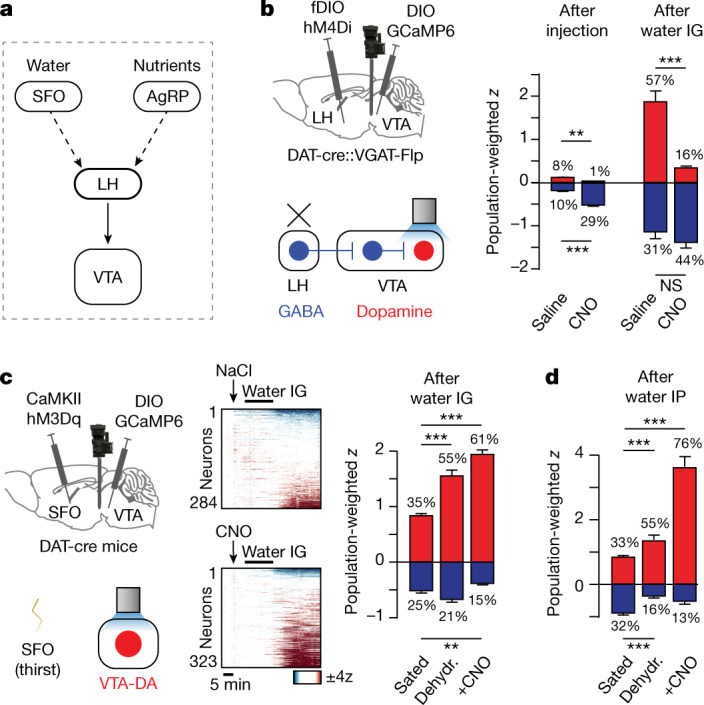
A forebrain–hypothalamic pathway that conveys internal state information to the VTA. **a**, A model of a possible anatomic pathway connecting interoceptive neurons for water and nutrients to the VTA. **b**, Left, schematic of simultaneous microendoscope imaging of VTA-DA neurons and chemogenetic inhibition (with hM4Di) of LH-GABA neurons. Right, population responses (as defined in Fig. 1) for VTA-DA neurons activated (red) or inhibited (blue) by saline or CNO, and after water infusion following saline or CNO. **c**, Left, schematic of simultaneous microendoscope imaging of VTA-DA neurons and chemogenetic activation (with hM3Dq) of SFO thirst neurons. Middle, dynamics of individual VTA-DA neurons in response to water infusion after saline or CNO injection in sated mice. Right, population responses of VTA-DA neurons activated (red) or inhibited (blue) after intragastric infusion. **d**, Population responses of VTA-DA neurons activated (red) or inhibited (blue) after intraperitoneal injection of water. Dehydr., dehydrated. Data are mean ± s.e.m. Statistics are shown in Extended Data Table 2.

To test whether this circuit transmits fluid balance information to the VTA (Fig. 3a), we first measured how activation of LH-GABA→VTA neurons influences the activity of VTA-GABA neurons. Optogenetic stimulation of LH-GABA terminals in the VTA combined with simultaneous imaging of VTA-GABA neuron cell bodies revealed that LH-GABA→VTA neuron activation induces strong and prolonged inhibition in the majority of VTA-GABA neurons (Extended Data Fig. 5e). This suggests that the natural activation of LH-GABA neurons by water would be sufficient to modulate VTA dynamics.

To test whether LH-GABA neurons are necessary for VTA-DA neurons to track changes in fluid balance, we targeted the inhibitory designer receptor exclusively activated by designer drugs (DREADD) hM4Di to LH-GABA neurons while imaging VTA-DA neurons. Infusion of water alone again caused strong activation of many DA neurons (Fig. 3b). Of note, silencing LH-GABA neurons before water intragastric infusion specifically blocked this subsequent VTA-DA neuron response (Fig. 3b). This suggests that LH-GABA neurons are the source of the signal that informs VTA-DA neurons about fluid balance (Fig. 3a).

**Extended Data Fig. 6:**
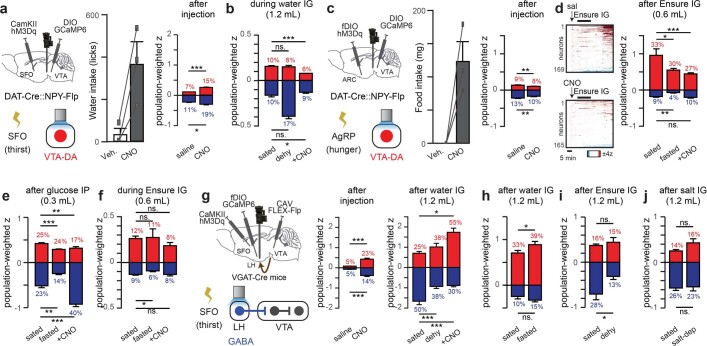
Hunger and thirst neurons control the gain of VTA-DA neuron responses to nutrients and water. **a**, (Left) Schematic for simultaneous microendoscope imaging of VTA-DA neurons and optogenetic activation of SFO “thirst” neurons. (Middle) Effect of SFO neuron activation on water intake (5 min in sated mice; n = 3 mice). (Right) Summary bar graphs and percentages of VTA-DA neurons activated (red) or inhibited (blue) by IP injection of saline or CNO. **b**, Mean responses and proportion of neurons activated and inhibited during IG infusion of water (1.2 mL) following SFO neuron activation, water deprivation, or neither. **c**, (Left) Schematic for simultaneous microendoscope imaging of VTA-DA neurons and optogenetic activation of AgRP “hunger” neurons. (Middle) Effect of AgRP neuron activation on food intake (5 min in sated mice; n = 3 mice). (Right) Mean responses and proportion of neurons activated and inhibited after AgRP neuron activation or control. **d**, (Left) Individual neuron dynamics. (Right) Mean responses and proportion of neurons activated and inhibited after IG infusion of Ensure (0.6 mL) following AgRP neuron activation, food deprivation, or neither. Note that both food deprivation and AgRP neuron stimulation counterintuitively reduce DA responses to IG nutrients. **e**, Mean responses and proportion of neurons activated and inhibited after intraperitoneal (IP) injection of 50% glucose (0.3 mL) following AgRP neuron activation, food deprivation, or neither. **f**, Mean responses and proportion of neurons activated and inhibited during IG infusion of Ensure (0.6 mL) following AgRP neuron activation, food deprivation, or neither. **g**, (Left) Schematic for simultaneous microendoscope imaging of LH-GABA→VTA neurons and chemogenetic activation (hM3Dq) of SFO “thirst” neurons. (Middle) Summary bar graphs and percentages of LH-GABA→VTA neurons activated (red) or inhibited (blue) by saline or CNO. (Right) Summary bar graphs and percentages of LH-GABA→VTA neurons activated (red) or inhibited (blue) after IG infusion of water (1.2 mL). **h-i**, Responses to food and water in an orthogonal need state. **h**, Mean responses and proportion of VTA-DA neurons activated and inhibited after IG infusion of water (1.2 mL) while sated or food-deprived. **i**, Mean responses and proportion of VTA-DA neurons activated and inhibited after Ensure IG infusion (1.2 mL) while sated or dehydrated. **j**, Mean responses and proportion of VTA-DA neurons activated and inhibited after IG infusion of hypertonic saline (0.3 M NaCl, 1.2 mL) in mice that are sated or sodium depleted. ns.P > 0.05, *P < 0.05, **P < 0.01, ***P < 0.001. All error bars show mean±s.e.m. Statistics in Extended Data Table 2.

Information about fluid balance enters the brain through dedicated interoceptive neurons including glutamatergic neurons in the subfornical organ (SFO) that are directly activated by dehydration^27^, drive thirst^38–43^, and are situated upstream of the LH in the thirst circuit^40^ (Fig. 3a). To investigate whether the SFO transmits fluid balance information to LH neurons and their VTA targets, we targeted the excitatory DREADD hM3Dq to thirst-promoting SFO neurons and imaged calcium responses in VTA-DA neurons. Injection of clozapine *N*-oxide (CNO) caused sated mice to drink voraciously, confirming SFO neuron activation, but CNO had only a subtle effect on VTA-DA neuronal activity at baseline (in the absence of water; Extended Data Fig. 6a), indicating that the SFO is not sufficient to drive VTA dopamine dynamics. By contrast, CNO potentiated the activation of VTA-DA neurons by subsequent administration of water, mimicking the effect of natural dehydration (Fig. 3c,d and Extended Data Fig. 1q). Similar to the SFO, stimulation of hunger-promoting AgRP neurons^44–46^ did not affect DA neuron activity at baseline but did modulate post-absorptive responses to internal nutrients (mimicking the effect of food deprivation; Extended Data Fig. 6c–f). Together, this indicates that SFO and AgRP neurons gate the magnitude of DA neuron responses to relevant changes in internal state but do not directly relay information about systemic hydration and nutrients.

To test whether this modulatory effect of SFO neurons on the VTA is conveyed through the LH, we again targeted hM3Dq to thirst-promoting SFO neurons and then imaged calcium dynamics in LH-GABA→VTA neurons. Chemogenetic stimulation of SFO neurons potentiated the activation of LH-GABA neurons by intragastric water infusion, similar to the effect of natural dehydration (Extended Data Fig. 6g) and as observed in VTA-DA neurons (Fig. 3c,d). Activating SFO neurons alone also caused a small but significant modulation of LH-GABA neuron activity at baseline (Extended Data Fig. 6g). Together, these findings reveal that SFO neurons modulate dopamine responses to water, at least in part, through effects on upstream LH-GABA neurons (Fig. 3a).

## Dopamine release at stages of ingestion

**Fig. 4:**
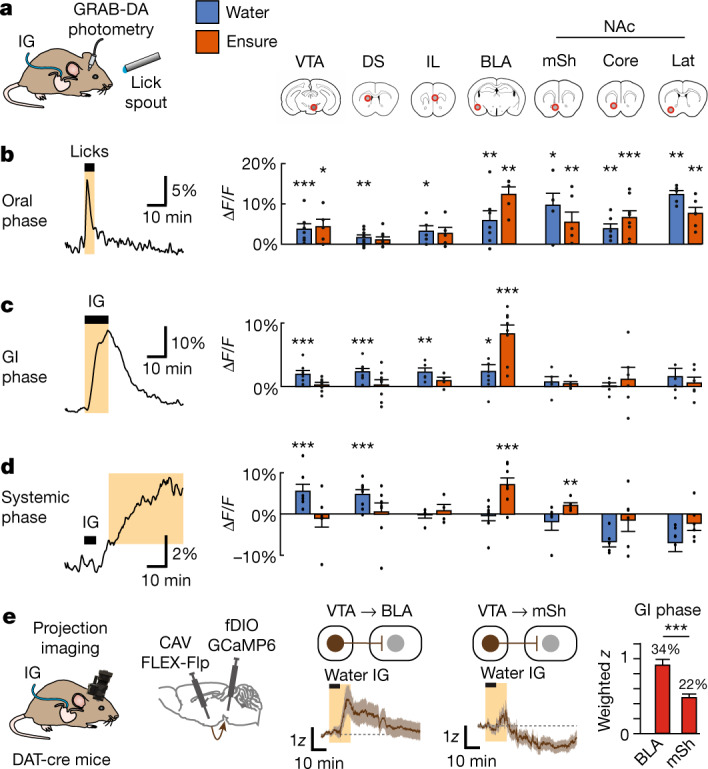
Dopamine release at different VTA targets tracks different stages of ingestion. **a**, Schematic showing the setup and seven recording sites (red circles) for measuring dopamine release. **b**, Left, example recording from the mSh showing dopamine release during the oral phase of water ingestion (30 s after the start of licking). Right, mean dopamine release in each recorded region during this oral phase during water (blue) and Ensure (orange) consumption. **c**, Left, example recording from BLA showing dopamine release during gastrointestinal phase for Ensure (first 12 min after intragastric infusion starts). Right, mean dopamine release in each recorded region during this gastrointestinal phase during water and Ensure infusion. **d**, Left, example recording from VTA showing dopamine release during systemic phase for water (12 to 50 min after the start of intragastric infusion). Right, mean dopamine release in each recorded region during this systemic phase following water and Ensure infusion. **e**, Left, schematic of projection-specific imaging. Middle, mean responses of VTA-DA→BLA neurons and VTA-DA→mSh neurons during water intragastric infusion (gastrointestinal phase highlighted). Right, mean responses (population-weighted *z*-score) of activated neurons projecting to BLA and mSh. *P < 0.05. Data are mean ± s.e.m. Statistics are shown in Extended Data Tables 2 and 3.

**Extended Data Fig. 7:**
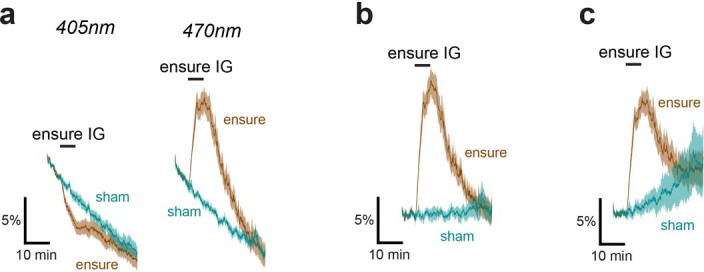
GRAB-DA signal normalization for long recordings. **a**, Mean GRAB-DA fluoresence responses in BLA during and after IG infusion of Ensure or sham infusion using 405 nm and 470 nm excitation wavelengths. Note the timescale of the recording. **b**, Mean GRAB-DA responses at 470 nm excitation when normalized to the 405 nm “isosbestic” response. **c**, Mean GRAB-DA responses at 470 nm excitation when normalized using an exponential fit to the fluorescence decay in the baseline period. Statistics in Extended Data Table 3.

**Extended Data Fig. 8:**
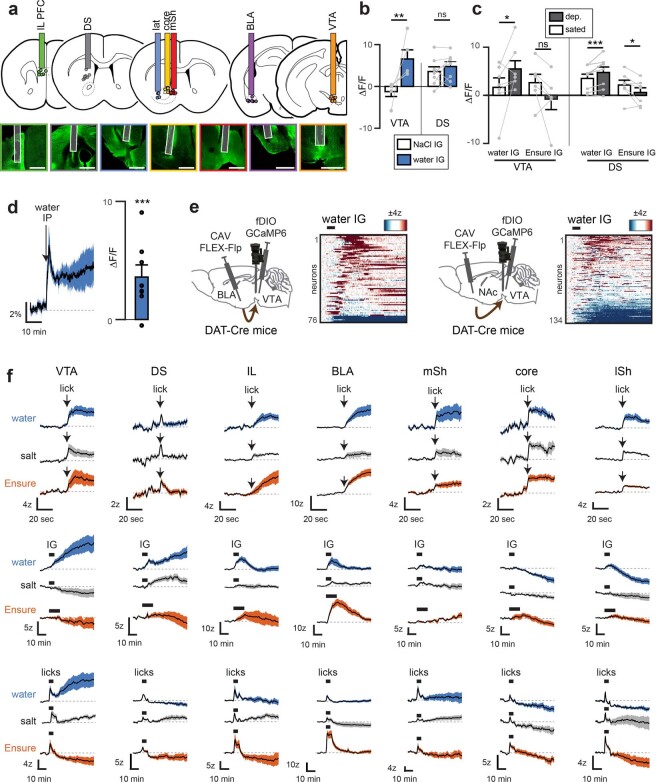
DA release in downstream targets in response to water, salt, and Ensure during different stages of ingestion. **a**, Approximate placements (dots) of photometry fibers for GRAB-DA recordings, alongside representative images. Scale bar, 1 mm. **b**, GRAB-DA fluorescence responses in VTA and DS to systemic hydration changes following IG infusion (1.2 mL) of water or hypertonic saline (0.3 M NaCl). **c**, GRAB-DA responses in VTA and DS following IG infusion (1.2 mL) of water and Ensure during sated state and following food or water deprivation. **d**, Mean GRAB-DA responses in VTA during and after IP injection of water. **e**, Dynamics of BLA and NAc projection neurons during and after IG infusion of water (see Fig. 4e). **f**, Mean GRAB-DA responses during self-paced drinking (top and bottom rows) and IG infusion (1.2 mL, middle row) of water (blue), 300 mM NaCl (grey), and Ensure (orange). ns.P > 0.05, *P < 0.05, **P < 0.01, ***P < 0.001. All error bars and shaded lines show mean±s.e.m. Statistics in Extended Data Table 2, 3.

To investigate how information about fluids and nutrients is transmitted from the VTA to downstream circuits, we prepared mice for intragastric infusion and fibre photometry recording of extracellular dopamine using GRAB-DA^5^ (Fig. 4a and Extended Data Fig. 7) in six densely innervated projection targets of VTA-DA neurons^36^—the infralimbic prefrontal cortex (IL), the basolateral amygdala (BLA), the dorsal striatum (DS), and three subdivisions of the nucleus accumbens (NAc) (lateral (lat), medial shell (mSh) and core). We also prepared mice for recordings in the VTA itself to measure local dopamine release^47–49^. We then measured dopamine release at these seven sites (Extended Data Fig. 8a) in response to an array of stimuli associated with ingestion, including self-paced eating and drinking as well as intragastric infusions of nutrients and fluids.

This revealed that dopamine is released at different brain sites in a way that tracks the passage of nutrients and fluids through stages of ingestion (from the mouth to gastrointestinal tract to blood; Fig. 4b–d and Extended Data Table 3). We found that oral nutrients and fluids triggered dopamine release in many brain regions that was time-locked to licking of food and water, including in NAc, as shown previously^4,5^. Subsequent detection of water in the gastrointestinal tract was represented in multiple areas but not the NAc, whereas gastrointestinal nutrients triggered dopamine release in BLA alone (Fig. 4c). Of note, dopamine release in BLA ramped up over tens of seconds during drinking in a manner more consistent with gastric filling than immediate orosensory feedback (Extended Data Fig. 8f). We confirmed with projection-specific imaging that the differential dopamine release in NAc and BLA in response to gastrointestinal signals matches the cell body calcium dynamics of the VTA-DA neurons that project to these two areas (Fig. 4e and Extended Data Fig. 8e).

In addition to these oral and gastrointestinal responses, we observed a third, post-absorptive stage that was characterized by prolonged dopamine release in the VTA itself (Fig. 4d). This (presumably somatodendritic^47–49^) dopamine release gradually ramped up following either self-paced drinking or intragastric water infusion, remained elevated for tens of minutes, and was potentiated by prior water deprivation (Extended Data Fig. 8c). Dopamine release in VTA was also observed (but with a more rapid onset) following intraperitoneal injection of water (Extended Data Fig. 8d), and the time course of dopamine release by VTA-DA neurons in response to water administered by these various routes mirrored the calcium dynamics of a large subpopulation of VTA-DA neurons (Fig. 1), which in turn tracked systemic rehydration. In addition to the VTA, we also observed dopamine release in DS during and after intragastric infusion of water (Fig. 4c,d and Extended Data Fig. 8b,c,f). Together, these findings reveal that the dopamine system is organized such that different stages of ingestion preferentially trigger dopamine release in distinct subsets of downstream targets.

## Role of dopamine in learning about fluids

Many animals obtain their water from diverse sources, including foods^9–15^, and therefore must learn which foods and fluids are rehydrating through ingestive experience. We considered the possibility that the activation of dopamine neurons by systemic rehydration could be involved in this learning process. Of note, although water rewards are widely used to train animals^50^, attempts to bypass normal drinking and drive operant behaviour with intragastric fluids alone have been unsuccessful^51,52^. However, we reasoned that changes in fluid balance might be more efficient at driving learning about oral cues such as flavours, since these two modalities are tightly coupled during normal ingestion.

**Fig. 5:**
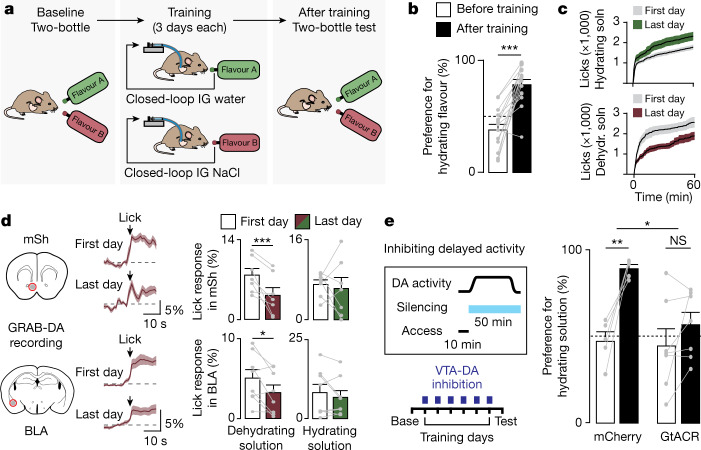
Post-ingestive changes in fluid balance drive learning about fluids via VTA-DA neurons. **a**, Schematic of closed-loop system for fluid preference training. Mice are hydrated by consumption of one flavoured solution (flavour A) and mildly dehydrated by another (flavour B) via intragastric infusion of water or saline that is triggered by licking. **b**, Preference for flavour A before and after training. **c**, Changes in total consumption on the first and last days of training. **d**, Left, GRAB-DA fluorescence in mSh and BLA during consumption of the dehydrating solution on first and last training days. Right, summary plots of lick-triggered GRAB-DA responses on first and last training days with each solution. **e**, Inhibiting VTA-DA neurons during training selectively after water access has been removed (minutes 10–60) eliminates preference learning in mice expressing GtACR but not in mCherry controls. Data are mean ± s.e.m. Statistics are shown in Extended Data Table 4.

To test this idea, we developed a closed-loop system for coupling licking to intragastric infusion, so that the flavour and rehydrating ability of an ingested solution could be independently varied (Fig. 5a; inspired by previous studies of nutrient flavour conditioning^8,22,53–56^). We programmed this system such that each lick of one flavoured solution triggered intragastric infusion of an equal volume of saline, and each lick of a differently flavoured solution triggered intragastric infusion of an equal volume of water. As a result, consumption of only one solution is rehydrating, but this cannot be initially anticipated on the basis of taste.

**Extended Data Fig. 9:**
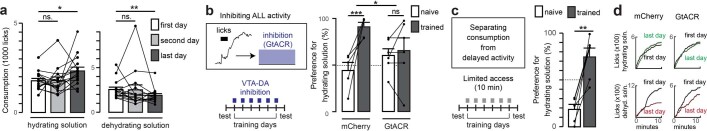
Timing of fluid preference learning. **a**, Summary plots of consumption of each solution on each fluid preference training day using the protocol in which mice have access to water for 60 min in each training day (see Fig. 5c). **b**, Illumination of VTA-DA neurons throughout the entire training session (60 min) prevents preference learning in mice expressing GtACR but not in the mCherry-expressing controls. **c**, (Left) Revised training protocol in which water access is removed after 10 min of consumption in each training session. (Right) Robust preference learning occurs even with only 10 min access (i.e. water is removed before VTA-DA activity has changed due to post-absorptive changes; n = 6). **d**, Changes in total consumption on the first and last days of training in delayed silencing experiment (Fig. 5e). ns.P > 0.05, *P < 0.05, **P < 0.01. All error bars and shaded lines show mean±s.e.m. Statistics in Extended Data Table 4.

**Extended Data Fig. 10:**
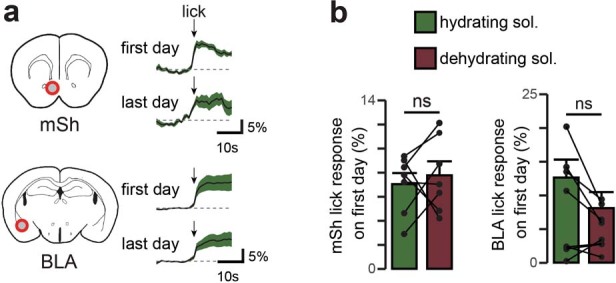
Dynamics of dopamine release during fluid preference training. **a**, GRAB-DA fluorescence in mSh and BLA during consumption of the hydrating solution on first and last training days (see Fig. 5e). **b**, Summary plots of lick-triggered GRAB-DA responses on first training days with each solution. ns.P > 0.05. All error bars and shaded lines show mean±s.e.m. Statistics in Extended Data Table 3.

We first determined preferences at baseline by giving mice a two-bottle test with these flavours in the absence of intragastric infusion. Mice were then trained by providing isolated access to each solution (which now triggered intragastric infusion) for three one-hour sessions on consecutive days. After training, the mice were tested again with a two-bottle test in the absence of infusion to measure any learned changes in preference. This revealed that mice learn to strongly prefer solutions paired with water infusion, as measured by two-bottle test (Fig. 5b and Extended Data Table 4) and consumption during training (Fig. 5c and Extended Data Fig. 9a), which was accompanied by changes in consumption-triggered dopamine release in both NAc and BLA (Fig. 5d and Extended Data Fig. 10). This indicates that slow and delayed changes in fluid balance can drive robust learning and changes in dopamine release, when paired with an appropriate oral cue.

To test whether DA neuron activity is necessary for this learning process, we used the inhibitory opsin stGtACR2 to silence VTA-DA neurons throughout training, which blocked the acquisition of fluid preference (Extended Data Fig. 9b). However VTA-DA neurons are activated by both oral and post-ingestive signals (Fig. 1). To test specifically the function of post-ingestive DA neuron activity, we modified the training protocol so that mice had access to each fluid for only 10 min per training session (thereby separating in time water consumption from its post-absorptive effects, which begin approximately 10 min after the start of drinking) (Extended Data Fig. 1d). Mice robustly learned to prefer the flavour associated with water infusion using this new protocol (Extended Data Fig. 9c), which enabled us to test the role of post-ingestive signals by optogenetically silencing VTA-DA neurons only after water access had been removed. Silencing of VTA-DA neurons during this post-absorptive phase was sufficient to block the acquisition of learned preference (Fig. 5e). This reveals that the activation of VTA-DA neurons by systemic hydration is necessary for learning the consequences of fluid ingestion, even when this activation occurs after consumption has ceased.

## Summary

Here we have shown that embedded within the mesolimbic dopamine system are multiple subsystems that track the internal, post-ingestive consequences of eating and drinking. We found that changes in fluid balance have particularly strong effects on DA neuron calcium dynamics, which led us to investigate how hydration signals are communicated to the dopamine system and used for learning. This revealed that GABAergic neurons in the LH relay information about systemic hydration to the VTA, whereas thirst-promoting neurons in the SFO control the gain of this signal. We further showed that dopamine is released in different downstream sites in response to detection of food and fluids at progressive stages of ingestion. We probed the function of these signals using a behavioural paradigm that uncouples the oral and systemic effects of ingested fluids, which revealed that post-ingestive rehydration alone can drive robust learning and that this requires VTA-DA neurons. This exposes an organizational logic whereby ingestion of food and water and their subsequent effects on internal state are differentially represented in the dopamine system and used for learning.

Previous studies of the dopamine system and fluids have focused on drinking and acute responses to water cues^4,19,57,58^. We examined changes in fluid balance over longer timescales and found that systemic dehydration and rehydration trigger the inhibition and activation, respectively, of many VTA-DA neurons. These responses track changes in blood osmolality, persist for tens of minutes, are independent of the method of fluid administration, and correlate with local dopamine release in the VTA. The function of somatodendritic dopamine release is poorly understood but is spatially localized, controlled by unique firing patterns and synaptic machinery^47,49^, and has the potential to gate the activity of specific DA neuron subsets and inputs^47,59,60^. Together these findings reveal that changes in fluid balance modulate not only DA neuron responses to water cues^61^, but also directly modulate the system as a whole.

In contrast to fluids, several studies have reported that ingested nutrients trigger dopamine release in striatum^62–66^, and we detected a subtle increase in dopamine in NAc mSh (but not core or lateral NAc or DS) after nutrients were delivered to the stomach (Fig. 4d). However, we found unexpectedly that intragastric nutrients triggered the highest level of dopamine release in BLA during both the gastrointestinal and systemic phase (Fig. 4c,d). The BLA is well known for processing nutrient information^67^ and previous work has shown that BLA lesions^68^ or pharmacology^55^ can block flavour learning. These findings prioritize further investigation of the VTA→BLA pathway to understand how internal nutrient signals drive learning about food.

Learning about foods and fluids requires bridging multiple timescales of signals. The dopamine system is well known for its role in learning relationships between external cues and gustatory rewards^19,20^, such as the sight of water and its taste. However, the taste of water is itself a cue that predicts a delayed change in fluid balance, and whether and how animals learn such relationships has been unclear. Here we have shown that animals can learn to associate tastes with delayed bodily rehydration, which activates VTA-DA neurons, and that this delayed activity is necessary for learning. This identifies a key pathway that enables animals to learn which foods and fluids to consume in order to remain hydrated. Further investigation of this circuitry may reveal principles for how the brain bridges the gap between transient cues associated with ingestion and delayed physiologic effects.

## Methods

Experimental protocols were approved by the University of California, San Francisco Institutional Animal Care and Use Committee, following the NIH Guide for the Care and Use of Laboratory Animals.

### Mouse strains

Experiments used adult mice (>6 weeks old) of both sexes, housed in temperature- and humidity-controlled facilities with 12-h light–dark cycle, and ad libitum access to water and standard chow (PicoLab 5053). We obtained DAT-ires-cre (B6.SJL-Slc6a3^tm1.1(cre)Bkmn^, Jackson cat. no. 006660) and Vgat-ires-cre (Slc32a1^tm2(cre)Lowl^, Jackson #016962) mice from Jackson Labs. Vgat-ires-flp knock-in mice (B6.Cg-Slc32a1^tm2.1(flop)Hze^) and Npy-ires-flp knock-in mice (B6.Cg-Npy^tm1.1(flop)Hze^) from the Allen Institute for Brain Science. DAT-ires-cre knock-in mice were crossed with Vgat-ires-flp knock-in mice or with Npy-ires-flp knock-in mice to generate double mutants.

### Behaviour

For all behaviour and recordings, mice were placed in sound-isolated behavioural chambers (Coulbourn Habitest Modular System) exclusively during the dark cycle. The chambers were cleaned between experiments to remove any remaining olfactory cues from previous experiments. All mice were habituated for at least 1 h to the behavioural chamber (with recording equipment and intragastric catheter simultaneously attached) before being used for any experiments on subsequent days. Mice were additionally re-habituated to the behavioural chamber for 10–20 min at the beginning of each testing session (during which the LED used for imaging was turned on, to reduce any initial bleaching artifacts at the beginning of the recording).

For lick response experiments, mice were either water-deprived (for experiments using water or 300 mM NaCl consumption) or food-deprived (for experiments using Ensure consumption) for 24 h prior to the experiment. Mice were then given access to a lickometer containing the appropriate solution (water, Ensure, or 300 mM NaCl solutions) for a total of 5 min, which was defined as beginning with the first lick bout (to control for the fact that some mice had a longer latency to drink). Access was terminated by removing the bottle from the chamber, with care to prevent any remaining olfactory cues (for example, by cleaning any drops). Experiments measuring responses to consumption of 300 mM NaCl were performed only on naive mice, because animals can learn to avoid hypertonic solutions after a single experience^69^. All mice were habituated initially to the lickometer and recording setup by providing access to a bottle containing water for one hour with the microendoscope camera attached.

Intraperitoneal injections were performed on the right side of the mouse. For recording responses to intraperitoneal injections alone, recordings continued for 30 min following injection. For recording response modulation by DREADD activation, CNO injection was followed by subsequent intragastric infusion or intraperitoneal injection after 5 min (see below). We used the following doses as indicated in the figure legends: 1.2 ml water, 0.6 ml 50% glucose, 0.12 ml 3 M NaCl, and 1.2 ml 154 mM NaCl (isotonic saline). Mice were given a sham intraperitoneal injection one day before being used for these experiments to habituate the mice.

Intravenous injections were performed by injecting hypertonic saline (100 μl, 1 M NaCl) into the lateral tail vein, while the mouse was kept in a custom-made restraint system that allows for concurrent imaging. Mice were habituated for 30 min to the setup before the experiment began. For recording responses to intravenous injections, recordings continued for 30 min following injection. The animals were not anaesthetized during these experiments.

Intragastric infusions were delivered at a rate of 100 or 200 μl min^−1^ using a syringe pump (Harvard Apparatus 70–2001), for 6 or 12 min (resulting in either 0.6 or 1.2 ml total infused volume). Solutions of NaCl (154 or 300 mM), glucose (25%) and Ensure were prepared using deionized water. We previously measured the latency for fluids to pass through the intragastric catheter itself as approximately 13 s at an infusion rate^20^ of 200 μl min^−1^. For infusion experiments, the intragastric catheter was attached to the syringe pump using plastic tubing and adapters (AAD04119, Tygon; LS20, Instech) before mice were placed into the behavioural chamber.

Vagal defferentation was performed by injecting mice prepared for microendoscopic imaging of VTA-DA neurons (see below section) with 50 mg kg^−1^ capsaicin. Mice were kept under anaesthesia with isoflurane for 1 h starting immediately before injection to avoid unnecessary pain, according to previously published protocols^70^. Efficacy of deafferentation was determined by testing number of eye wipes in the 15 s after ocular delivery of 0.1% capsaicin (compared with mice which underwent the same deafferentation surgery but received a saline injection; Extended Data Fig. 5f).

### Intragastric catheterization

Intragastric catheters were installed following our published protocols^20,24^. In brief, sterile access veterinary catheters (C30PU-RGA1439, Instech Labs) were attached to sterile vascular access buttons (VABM1B/22, 22, Instech Labs). Mice were anaesthetized with ketamine-xylazine, the catheter was surgically implanted into the avascular stomach, and the port on the access button was secured to the mouse’s back (with the port facing out, that is, dorsally). A protective aluminium cap (VABM1C, Instech Labs) was placed over the port to protect it between experiments. Mice were allowed one week to recover following intragastric surgery before the start of experiments.

### Preparation of mice for microendoscopy

Mice were prepared for microendoscope recordings using the general procedures we have reported^20^. In brief, in the first surgery mice were injected with an adeno-associated virus (AAV) expressing a GCaMP and a GRIN lens was lowered into the brain. Four weeks later, mice underwent a second surgery in which a baseplate (Inscopix 100-000279) was placed above the lens and affixed with adhesive cement (MetaBond) and then covered after surgery with a baseplate cover (Inscopix 100-000241). Most animals then underwent a third surgery to install an intragastric catheter for fluid infusions into the stomach. The details for each cohort are below.

#### Microendoscope imaging of VTA-DA neurons

DAT-cre mice (*n* = 14) were prepared for imaging by injecting AAV5-Syn-FLEX-GCaMP6f-WPRE-SV40 (300 nl; 7.2 × 10^12^ viral genome copies (vg) ml^−1^; Addgene) into the left VTA (−3.1 mm AP, +0.5 mm ML, −4.5 mm DV) and installing a GRIN lens (6.1 × 0.5 mm; Inscopix 1050-004610) 0.15 mm above the injection site in the same surgery. In subsequent surgeries, each mouse was baseplated and then equipped with an intragastric catheter.

#### Microendoscope imaging of LH-GABA neurons

VGAT-cre mice (*n* = 5) were prepared for imaging by injecting AAV5-Syn-FLEX-GCaMP6f-WPRE-SV40 (300 nl; 7.2 × 10^12^ vg ml^−1^; Addgene) into the left LH (−1.5 mm AP, +1.0 mm ML, −5.1 mm DV) and installing a GRIN lens (8.3 × 0.5 mm; Inscopix 1050-004611) 0.15 mm above the injection site in the same surgery. In subsequent surgeries, each mouse was baseplated and then equipped with an intragastric catheter

#### Microendoscope imaging of LH-GABA→VTA neurons

VGAT-cre mice (*n* = 3) were prepared for imaging by injecting AAV1-Ef1a-fDIO-GCaMP6m (300 nl; 2 × 10^12^ vg ml^−1^; Janelia Vector Core) into the left LH (−1.5 mm AP, +1.0 mm ML, −5.1 mm DV), CAV2-Flx-Flp (300 nl; 6 × 10^12^ vg ml^−1^; Plateforme de Vectorologie) into left VTA (−3.1 mm AP, +0.5 mm ML, −4.5 mm DV) and installing a GRIN lens (8.3 × 0.5 mm; Inscopix 1050-004611) 0.15 mm above the LH injection site. In subsequentsurgeries, each mouse was baseplated and then equipped with an intragastric catheter.

#### Microendoscope imaging of VTA-GABA neurons and simultaneous optogenetic activation of LH-GABA neuron terminals

VGAT-cre mice (*n* = 3) were prepared for imaging by injecting AAV5-hSyn-DIO-ChrimsonR-tdTomato (300 nl; 4.4 × 10^12^ vg ml^−1^; Addgene) in the left LH (−1.5 mm AP, +1.0 mm ML, −5.1 mm DV); AAV5-Syn-FLEX-GCaMP6f-WPRE-SV40 (300 nl; 7.2 × 10^12^ vg ml^−1^; Addgene) in the left VTA (−3.1 mm AP, +0.5 mm ML, −4.5 mm DV) and installing a GRIN lens (6.1 × 0.5 mm; Inscopix 1050-004610) 0.15 mm above the injection site in the same surgery. To stimulate during recording, a 620 nm LED was delivered through the nVoke 2.0 camera (15 mW mm^−2^ LED power, 20 Hz pulse frequency, 1 ms pulse width, 2 s ON and 3 s OFF cycle).

#### Microendoscope imaging of VTA-DA neurons and simultaneous LH-GABA neuron inhibition

DAT-cre::VGAT-Flp mice (*n* = 6) were prepared for imaging by injecting AAVDJ-hSyn-fDIO-hM4Di-mCherry (400 nl; 1.1 × 10^13^ vg ml^−1^; Stanford) bilaterally into the LH (−1.5 mm AP, ± 1.0 mm ML, −5.1 mm DV); AAV5-Syn-FLEX-GCaMP6f-WPRE-SV40 (300 nl; 7.2 × 10^12^ vg ml^−1^; Addgene) into the left VTA (−3.1 mm AP, +0.5 mm ML, −4.5 mm DV); and installing a GRIN lens (6.1 × 0.5 mm; Inscopix 1050-004610) 0.15 mm above the injection site in the same surgery. In subsequent surgeries, each mouse was baseplated and then equipped with an intragastric catheter.

#### Microendoscope imaging of VTA-DA neurons and simultaneous SFO-Glut neuron activation

DAT-cre mice (*n* = 3) were prepared for imaging by injecting AAV2-CamKIIa-HA-hM3Dq-ires-mCitrine (100 nl; 2 × 10^12^ vg ml^−1^; UNC Vector Core) into the SFO (−0.6 mm AP, 0 mm ML, −2.8 mm DV); AAV5-Syn-FLEX-GCaMP6f-WPRE-SV40 (300 nl; 7.2 × 10^12^ vg ml^−1^; Addgene) into the left VTA (−3.1 mm AP, +0.5 mm ML, −4.5 mm DV); and installing a GRIN lens (6.1 × 0.5 mm; Inscopix 1050-004610) 0.15 mm above the injection site in the same surgery. To confirm functional expression of hM3D, mice were injected with either CNO or saline (100 μl) and then were given access to water for 5 min (Extended Data Fig. 6a). In subsequent surgeries, each mouse was baseplated and then equipped with an intragastric catheter

#### Microendoscope imaging of VTA-DA neurons and simultaneous AgRP neuron activation

DAT-cre::NPY-Flp mice (*n* = 3) were prepared for imaging by injecting AAV1-Ef1a-fDIO-hM3Dq-mCherry (200 nl; 3 × 10^12^ vg ml^−1^; Stanford) into the ARC bilaterally (−1.75 mm AP, ± 0.2 mm ML, −5.9 mm DV); AAV5-Syn-FLEX-GCaMP6f-WPRE-SV40 (300 nl; 7.2 × 10^12^ vg ml^−1^; Addgene) into the left VTA (−3.1 mm AP, +0.5 mm ML, −4.5 mm DV), and installing a GRIN lens (6.1 × 0.5 mm; Inscopix 1050-004610) 0.15 mm above the injection site in the same surgery. To confirm functional expression of hM3D, mice were injected with CNO or saline (100 μl) and consumption of Ensure was recorded for 5 min (Extended Data Fig. 6c). In subsequent surgeries, each mouse was baseplated and then equipped with an intragastric catheter

#### Microendoscope imaging of LH-GABA→VTA neurons and simultaneous SFO-Glut neuron activation

VGAT-cre mice (*n* = 4) were prepared for imaging by injecting AAV2-CamKIIa-HA-hM3Dq-ires-mCitrine (100 nl; 2 × 10^12^ vg ml^−1^; UNC Vector Core) into the SFO (−0.6 mm AP, 0 mm ML, −2.8 mm DV), AAV1-Ef1a-fDIO-GCaMP6m (300 nl; 2 × 10^12^ vg ml^−1^; Janelia Vector Core) into the left LH (−1.5 mm AP, +1.0 mm ML, −5.1 mm DV), and CAV2-Flx-Flp (300 nl; 6 × 10^12^ vg ml^−1^; Plateforme de Vectorologie) into left VTA (−3.1 mm AP, +0.5 mm ML, −4.5 mm DV). A GRIN lens (8.3 × 0.5 mm; Inscopix 1050-004611) was implanted 0.15 mm above the LH injection site in the same surgery. In subsequent surgeries, each mouse was baseplated and then equipped with an intragastric catheter

#### Microendoscope imaging of VTA-DA→NAc mSh neurons

DAT-cre mice (n = 3) were prepared for imaging by injecting AAV1-Ef1a-fDIO-GCaMP6m (300 nl; 2 × 10^12^ vg ml^−1^; Janelia Vector Core) into the left VTA (−3.1 mm AP, +0.5 mm ML, −4.5 mm DV) and CAV2-Flx-Flp (100 nl; 6 × 10^12^ vg ml^−1^; Plateforme de Vectorologie) into left NAc mSh (+1.3 mm AP, +0.5 mm ML, −4.4 mm DV). A GRIN lens (6.1 × 0.5 mm; Inscopix 1050-004610) was implanted 0.15 mm above the VTA injection site in the same surgery. In subsequent surgeries, each mouse was baseplated and then equipped with an intragastric catheter

#### Microendoscope imaging of VTA-DA→BLA neurons

DAT-cre mice (*n* = 3) were prepared for imaging by injecting AAV1-Ef1a-fDIO-GCaMP6m (300 nl; 2 × 10^12^ vg ml^−1^; Janelia Vector Core) into the left VTA (−3.1 mm AP, +0.5 mm ML, −4.5 mm DV) and CAV2-Flx-Flp (200 nl; 6 × 10^12^ vg ml^−1^; Plateforme de Vectorologie) into left BLA (−1.8 mm AP, +2.9 mm ML, −4.7 mm DV). A GRIN lens (6.1 × 0.5 mm; Inscopix 1050-004610) was implanted 0.15 mm above the VTA injection site in the same surgery. In subsequent surgeries, each mouse was baseplated and then equipped with an intragastric catheter.

### Microendoscope recordings

Microendoscopy videos were acquired at 8 Hz (0.6–0.8 mW mm^−2^ 455 nm LED power, 8.0 gain, 2× spatial downsampling) using Inscopix software (Data Acquisition Software v. 151; https://support.inscopix.com/support/products/nvista-30-and-nvoke-20/data-acquisition-software-v151). After acquisition, videos were first pre-processed, spatially (binning factor of 2) and temporally (binning factor of 2) downsampled, put through a spatial bandpass filter (to remove noise and out-of-focus cells), and motion-corrected using Inscopix Data-Processing Software (v1.3.1, https://support.inscopix.com/support/products/data-processing-software/inscopix-data-processing-v131). Videos were removed if excessive motion (larger than the diameter of the average neuron in any direction) occurred that could not be addressed by additional motion-correction. Activity traces for individual neurons were then extracted from these videos using the constrained non-negative matrix factorization (CNMF-E) pipeline (http://www.github.com/zhoupc/cnmfe) implemented in MATLAB. After initial CNMF-E segmentation, extracted neurons were manually refined to avoid uncorrected motion artefacts, region of interest duplication and over-segmentation of the same spatial components. Neurons were removed if large bleaching artefacts (which fit a defined exponential decay function) occurred during the 10 min baseline period. For each experiment, activity traces for individual neurons were extracted from recordings from 3–6 mice and then pooled for subsequent analysis. We could readily align cells between recording sessions separated by two weeks. This relied on custom software combined with manual verification of all alignments.

Head acceleration was acquired using Inscopix software (Data Acquisition Software v. 151) from the 3-axis accelerometer built into the nVoke/nVista cameras, which provided *xyz* acceleration data at 50 Hz temporal resolution.

Fluid consumption was monitored with a capacitive lickometer and recorded using the nVista/nVoke (v. 3.0 nVista system; v. 2.0 nVoke system; https://support.inscopix.com/support/products/nvista-30/nvista-30) data acquisition systems and software (Data Acquisition Software v. 151; https://support.inscopix.com/support/products/nvista-30-and-nvoke-20/data-acquisition-software-v151) during microendoscope imaging experiments.

### Dopamine photometry

Wild-type mice (C57BL/6J, Jackson cat. no. 000664) were prepared for photometry recordings by injecting AAV9-hSyn-GRAB-DA1h (200 nl; 1.8×10^13^ vg ml^−1^; Janelia Vector Core) into the following sites: left VTA (*n* = 10 mice; −3.1 mm AP, +0.5 mm ML, −4.5 mm DV), BLA (*n* = 12 mice; −1.8 mm AP, +2.9 mm ML, −4.7 mm DV), IL PFC (*n* = 13 mice; +1.7 mm AP, +0.3 mm ML, −2.85 mm DV), DS (*n* = 8 mice; +1.5mm AP, +1.5mm ML, −3.5mm DV); NAc mSh (*n* = 14 mice; +1.3 mm AP, +0.5 mm ML, −4.4 mm DV), NAc core (*n* = 7 mice; +1.4 mm AP, +1.0 mm ML, −4.5 mm DV), or lateral NAc (*n* = 8 mice; +1.0 mm AP, +1.75 mm ML, −5.0 mm DV). In the same surgery, an optical fibre (0.4 mm inner diameter by 3.5, 5.4, or 6.3 mm length; Doric Lenses, MFC_400/430-0.48) was installed 0.1 mm above the injection site. After allowing at least one week to recover from intracranial surgery, mice then underwent a second surgery to install an intragastric catheter as described above.

For GRAB-DA signal acquisition, implanted mice were tethered to a patch cable (Doric Lenses, MFP_400/460/900-0.48_2m_FCM-MF2.5). Continuous 6 mW blue LED (470 nm) and UV LED (405 nm) served as excitation light sources. These LEDs were driven by a multichannel hub (Thorlabs), modulated at 211 Hz and 511 Hz respectively, and delivered to a filtered minicube (Doric Lenses, FMC6_AE(400–410)_E1(450–490)_F1(500–540)_E2(550–580)_ F2(600–680)_S) before connecting through optic fibres (Doric Lenses, MFC_400/430-0.48). GRAB-DA GFP signals and UV isosbestic signals were collected through these same fibres into a femtowatt silicon photoreceiver (Newport, 2151). Digital signals were sampled at 1.0173 kHz, demodulated, lock-in amplified, sent through a processor (RZ5P, Tucker-Davis Technologies), and collected by Synapse (TDT). After the experiment, these data was exported via Browser (TDT), and in MATLAB temporally downsampled to 4 Hz. All plotted photometry traces in this paper are an average of two replicate traces for the same mouse (performed within 2 weeks of each other), with the exception for 300 mM NaCl consumption which can only be tested once when mice are naive. In some cases, mice were removed from the study due to health issues before they could be tested with all stimuli, and as a result some pairwise comparisons are not possible for every mouse in the cohort.

Fluid consumption was also monitored with an electrical lickometer and recorded using software Synapse (TDT), and afterwards exported via Browser (TDT), and analysed in MATLAB.

For GRAB-DA photometry recording during fluid preference training, access to the flavoured solutions was restricted during a baseline period of 10 min at the beginning of the recording (this was in addition to the 10–20 min acclimatization period previously mentioned).

### Chemogenetics

We prepared mice for in vivo chemogenetic activation or inhibition as described above. CNO (1 mg kg^−1^, 100 μl) or vehicle (0.6% DMSO in saline) was delivered by intraperitoneal injection.

### Fluid preference training

Mice were continuously water restricted by providing 1 ml of water per day (in addition to water obtained during testing and training) and were removed from the study if their body weight fell below 85% of the initial body weight. Mice were tested and trained using a ten-day protocol based on similar procedures for nutrient flavour conditioning^22^. In brief, the initial preference for the solutions was determined using a two-bottle test over the first two days. Training occurred over the next six days, after which preference was reassessed. Mice were acclimatized to the behavioural chamber for 10–20 min before flavoured solutions became accessible. Approximately equal number of female and male mice were used for all fluid preference training experiments. Catheters for intragastric infusion were attached to the mice during all experiments (including two-bottle tests). Mice were taken off water restriction immediately after the ten-day training protocol and were allowed one week to recover.

During training, mice were given isolated access to one of two flavoured solutions, which triggered intragastric infusion when consumed. Mice were separated out into four groups. For the first three days of training, the first group was given 1 h access to one solution containing 0.05% unsweetened Grape Kool-Aid (Kraft Foods) mixed with 0.025% Saccharin. Consumption of this solution, measured using an electrical lickometer, triggered 1 μl intragastric infusion of 600 mM NaCl. For the next three consecutive days, the solution was replaced with a solution containing 0.05% unsweetened Lemon-Lime Kool-Aid (Kraft Foods) mixed with 0.025% Saccharin, which triggered 1 μl intragastric infusion of water when consumed. The second group went through the same training, but the order of the flavours was reversed. The third and fourth group went through the same protocols as groups one and two respectively, but instead grape was associated with water infusion and lime was associated with 600 mM NaCl infusion. In brief, the groups were as follows. Group 1: grape→NaCl infusion (days 1–3); lime→water infusion (days 4–6); group 2: lime→water infusion (days 1–3); grape→NaCl infusion (days 4–6); group 3: grape→water infusion (days 1–3); lime→NaCl infusion (days 4–6); group 4: lime→NaCl infusion (days 1–3); grape→water infusion (days 4–6).

Initial and acquired preferences were determined using two-bottle tests on two subsequent days before and after training (four days total). Flavoured solution placement (front or back of the cage) was randomized on the first day and reversed on the second day of the two-bottle test.

For two-bottle tests, mice were given 1 h access to two solutions containing 0.05% unsweetened Kool-Aid, either lemon-lime or grape (Kraft Foods), mixed with 0.025% Saccharin. Preference for the lime solution was calculated using the formula (lime licks/total licks). Fluid consumption was monitored with an electrical lickometer and recorded using software Synapse (TDT), and afterwards exported via Browser (TDT), and analysed in MATLAB.

For some experiments (Fig. 5e and Extended Data Fig. 9c) access was given for a limited time during training. These experiments were done according to the same protocol above, but access to the flavoured solution was removed 10 min after the first lick during each training day.

For VTA-DA silencing throughout fluid preference training (Extended Data Fig. 9b), AAV8-hSyn-DIO-stGtACR2-fRED (400 nl, 7.9 × 10^12^ vg ml^−1^; Stanford) or AAV5-Ef1a-DIO-mCherry (400 nl, 7.3 × 10^12^ vg ml^−1^; UNC Vector Core) was injected into the VTA bilaterally (−3.1 mm AP, ± 0.5 mm ML, −4.5 mm DV) and an optical fibre with a 200-μm inner diameter (Thorlabs FT200UMT, CFLC230-10) was placed 0.30 mm above and between the injection sites (−3.1 mm AP, 0 mm ML, −4.2 mm DV) in the same surgery in DAT-cre mice (*n* = 11 mice). During the training period, a DPSS 473-nm laser (Shanghai Laser and Optics Century BL473-100FC) was controlled by Graphic State software (v.4.2, http://www.coulbourn.com/category_s/363.html) through a TTL signal generator (Coulbourn H03-14) and synchronized with bottle availability. The laser power was measured to be ~1–2 mW at the patch cable tip and was delivered continuously for the experimental hour.

For delayed VTA-DA silencing during fluid preference training (Fig. 5e), AAV8-hSyn-DIO-stGtACR2-fRED (400 nl, 7.9 × 10^12^ vg ml^−1^; Stanford) or AAV5-Ef1a-DIO-mCherry (400nl, 7.3 × 10^12^ vg ml^−1^; UNC Vector Core) was injected into the VTA bilaterally (−3.1 mm AP, ± 0.5 mm ML, −4.5 mm DV), and two optical fibres with a 200-μm inner diameter (Thorlabs FT200UMT, CFLC230-10) were placed at angle 100 μm away from the 2 target sites in the same surgery in DAT-cre mice (*n* = 12 mice). During the training period, a DPSS 473-nm laser (Shanghai Laser and Optics Century BL473-100FC) was turned on 10 min after the first lick during each training day. Immediately prior to the laser turning on, access to the solution was removed, such that silencing did not overlap with consumption. The laser power was measured to be ~1–2 mW at the patch cable tip and was delivered continuously for the remainder of the experimental hour.

### Plasma osmolality measurements

Mice received an intraperitoneal injection (1.2 ml water or 0.12 ml 3 M NaCl). Blood (250 μl) was collected EDTA-coated capillary tubes (RAM Scientific 07-6011) from the right cheek 5 min before the injection and from the left cheek at a specific timepoint following the injection (0 min, 5 min, 10 min, 15 min, 20 min, 25 min or 30 min). The blood collection process took on average 30 s per mouse (samples were thrown out if collection took over 2 min). The blood was centrifuged (1,000*g* for 30 min), and the supernatant was removed and again centrifuged (10,000*g* for 30 min) to isolate plasma. Plasma quality was graded according to colour and samples were thrown out with a lightness under 70% (see the colour #ff6666 for reference). Plasma osmolality was immediately measured in duplicate for each sample using a freezing-point osmometer (Fiske Associates 210). The difference between the osmolalities of the two samples was used for analysis. Mice were allowed one week for recovery between samples.

### Histology

Mice were transcardially perfused with PBS followed by 10% formalin. Whole brains were dissected, and kept in 10% formalin overnight at 4 °C. The next day, the brains were transferred to 30% sucrose for overnight cryo-protection at 4 °C. Sections (40 μm) were prepared with a cryostat. To visualize fluorescent labelling, these sections were mounted with DAPI Fluoromount-G (Southern Biotech) and then imaged with the confocal microscope without staining. Images underwent minimal processing using ImageJ (v.1.53e).

### Data analysis

We analysed behaviour data, fibre photometry data and microendoscope imaging data using custom MATLAB (v.R2020b, http://www.mathworks.com/products/matlab) scripts.

For microendoscope imaging analysis, all responses were normalized using the function *z* = (*C*_raw_ − *μ*)/*σ*, where *C*_raw_ is an output of the CNMF-E pipeline, *μ* is mean *C*_raw_ during the baseline period (first 10 min) and *σ* is the standard deviation of *C*_raw_ during that same baseline period. For action potential analysis, the inferred spike rate (*S*; output of CNMF-E pipeline) was used. For baseline fluorescence analysis, *C*_raw_ was put through a 1/60 Hz lowpass filter (which removes all changes occurring faster than 1 min).

For photometry analysis, all responses were normalized using the function: Δ*F*/*F*_0_ = (*F* − *F*_0_)/*F*_0_, where *F* is the raw photometry signal and *F*_0_ is the fluorescence predicted using the signal obtained with 405 nm excitation (using a linear regression model of both signals during the 10 min baseline period; Extended Data Fig. 7a–c). For data presentation, plotted traces were additionally downsampled to 1 Hz (this was done to decrease the size of each graph).

For quantification of activity at different timescales of ingestion, certain epochs were defined for oral, gastrointestinal, and systemic stages of ingestion. These epochs were updated after analysing the rise times of each response in initial recordings. Neurons were considered activated during these epochs, if their mean change of activity during the epoch exceeded +1*z* (and, in the case of Extended Data Fig. 3c, was greater than the mean change of activity during other epochs).

Oral (lick) responses were defined for microendoscope recordings as the average *z*-scored change of activity in the 30 s following the start of the first licking bout. For GRAB-DA recordings, lick (oral) responses were defined as the average Δ*F*/*F*_0_ change of fluorescence in the 30 s following the start of the first licking bout. Throughout the paper, a drinking bout is defined as any set of licks lasting ten or more seconds in which no inter-lick interval is greater than two seconds. For fluid preference training, all lick bouts for a single mouse were averaged and treated as a single replicate.

Gastrointestinal responses were defined as average changes of activity occurring in the 12 min after the start of intragastric infusion (based on the length of most infusions in this paper, as well as rise time of systemic response).

Systemic (absorptive) responses were initially defined (Fig. 1) as average changes of activity occurring after solution access was removed or intragastric infusion and intraperitoneal injection ended. Based on subsequent analysis of rise time (10–14 after the start of drinking and intragastric infusion), and to ensure systemic responses were distinct from those during the gastrointestinal phase, systemic (absorptive) responses were redefined as average changes of activity occurring 12–32 min after the start of intragastric infusion or first lick bout for microendoscopic recordings (or 12–50 min after the start of intragastric infusion or first lick bout for photometry recordings). In both photometry and microendoscopic recordings, systemic responses were defined as 0–30 min after intraperitoneal injection. Rise time was calculated as the time it takes an activated or inhibited neuron to reach 50% of its peak activity change in a sigmoidal fit. A neuron was considered inhibited during a particular epoch if the average change of activity was more negative than −1*z*, and activated if the average change of activity was more positive than +1*z*.

Head acceleration was defined as the rectified sum of acceleration in all axes from the accelerometer positioned on the nVista/nVoke camera (see ‘Microendoscope recordings’). This summed signal was filtered at 5 Hz with a third-order zero-phase Butterworth filter (to remove transients). Acceleration below a threshold (defined by Otsu’s method) was used to distinguish movement from rest.

Throughout this Article, values are generally reported as mean ± s.e.m. (error bars or shaded area). In figures with simple linear regressions, shaded areas represent the 95% confidence interval for the line of best fit. Except for linear regressions, nonparametric tests were uniformly used. *P* values for paired and unpaired comparisons were calculated using two-sided permutation tests (10,000 iterations or exact when possible). *P* values for comparisons across multiple groups were initially assessed using ANOVA but reported as values from two-sided permutation tests. Occasionally, one-sided permutation tests were performed when a particular outcome was clearly expected from experiments done earlier in the paper (one-sided tests were used for Fig. 5e and Extended Data Fig. 9b, and throughout Fig. 4 and Extended Data Fig. 8). For the main results in Fig. 1, intra-class correlations^71^ (= variance between mice/total variance) and linear-mixed model effects were calculated (Extended Data Table 1) to ensure the effects were not due to outliers originating from one mouse. Power calculations were used to predetermine sample size when prediction of effect size was possible based on previous experiments (for example, a power calculation was used for Fig. 5e but not for Fig. 5b). The experiments were not randomized. The investigators were blinded to allocation during experiments and outcome assessment during the two VTA-DA silencing experiments.

### Reporting summary

Further information on research design is available in the Nature Research Reporting Summary linked to this paper.

## Online content

Any methods, additional references, Nature Research reporting summaries, source data, extended data, supplementary information, acknowledgements, peer review information; details of author contributions and competing interests; and statements of data and code availability are available at 10.1038/s41586-022-04954-0.

### Supplementary information

Reporting Summary

## Figures and Tables

**Extended Data Table 1:**
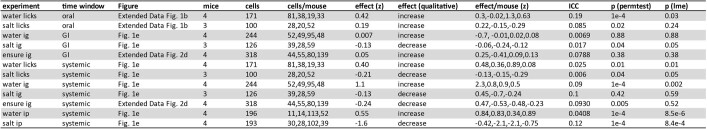
Linear Mixed-Effects Models (LME)

Table shows the results of linear-mixed models applied to the major findings in Figs. 1 and 2. Experiment, relevant Figure, number of mice recorded, number of cells recorded, and number of cells recorded per mouse (cells/mouse) are indicated alongside measures of activity, namely the mean z-scored change of activity for all recorded cells (Effect) and mean z-scored change of activity for all recorded cells in each mouse (Effect/mouse(z)). Statistical measurements of activity variance attributable to activity differences across mice (ICC, or Intra-class correlations), as well as p-values obtained using a permutation test (p(permtest)) or a linear mixed-effects model taking into account activity variance across mice (p(lme)) are also shown. Intra-class correlations were calculated using the equation varbetween mice/vartotal . Note that permutation tests used 10,000 permutations, making 1e-4 the lowest possible p-value obtainable by those tests. Time window indicates the phase of ingestion during which activity was quantified. The “oral” time window was defined as the 30 s immediately following the start of the first licking bout. The “GI” time window was defined as the 12 min immediately following the onset of IG infusion. The “Systemic” time window was defined as the time over which the infused, or injected solution would be absorbed into the blood (0–20 min after the end of IG infusion or self-paced consumption, 0–30 min after intraperitoneal injection).

**Extended Data Table 2:**
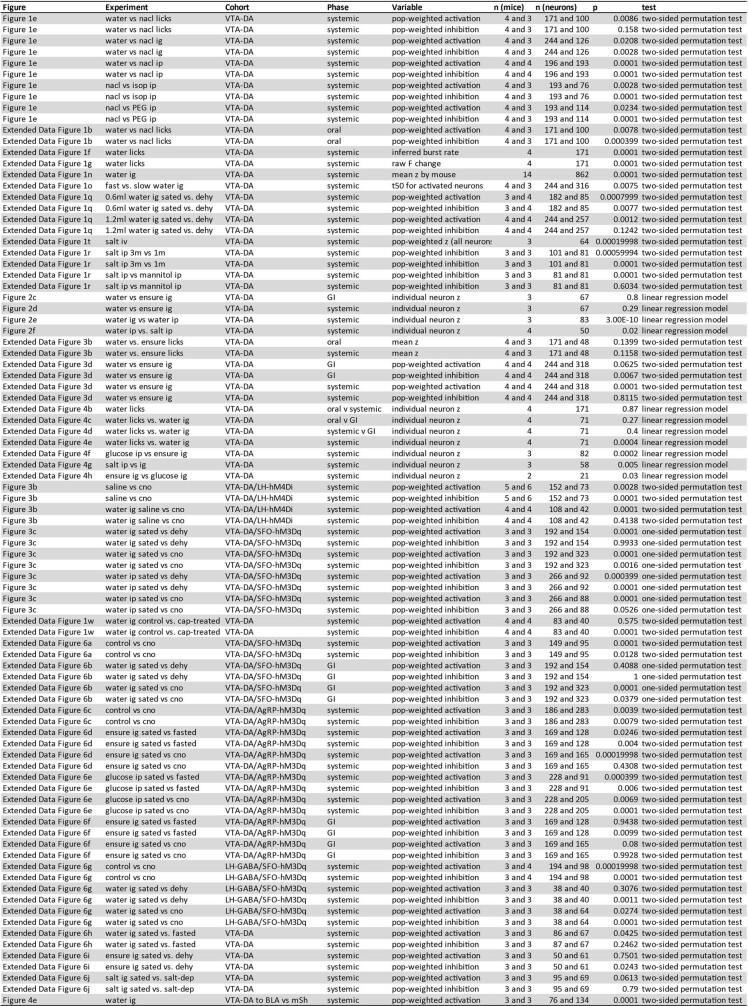
Statistics for single-cell recording data

Statistics are shown for each variable quantified during a given phase of an experiment in each figure. The number of mice (n(mice)) and neurons (n(neurons)) recorded in the experimental cohort are given alongside the p-value measured by the given statistical test. The phase is the time window during which activity was quantified. The “oral” time window was defined as the 30 s immediately following the start of the first licking bout. The “GI” time window was defined as the 12 min immediately following the onset of IG infusion. The “Systemic” time window was defined as the time over which the infused, or injected solution would be absorbed into the blood (12–32 min after the onset of IG infusion or 0–20 min after the end of self-paced consumption, 0–30 min after intraperitoneal injection). Note that permutation tests used 10,000 permutations, making 1e-4 the lowest possible p-value obtainable by those tests. Limitations in cross-registration for individual neurons resulted in number of mice and neurons differing from previous figures. One-sided tests were only used when an effect direction could be clearly predicted based on previous experiments. No corrections were made for multiple comparisons.

**Extended Data Table 3:**
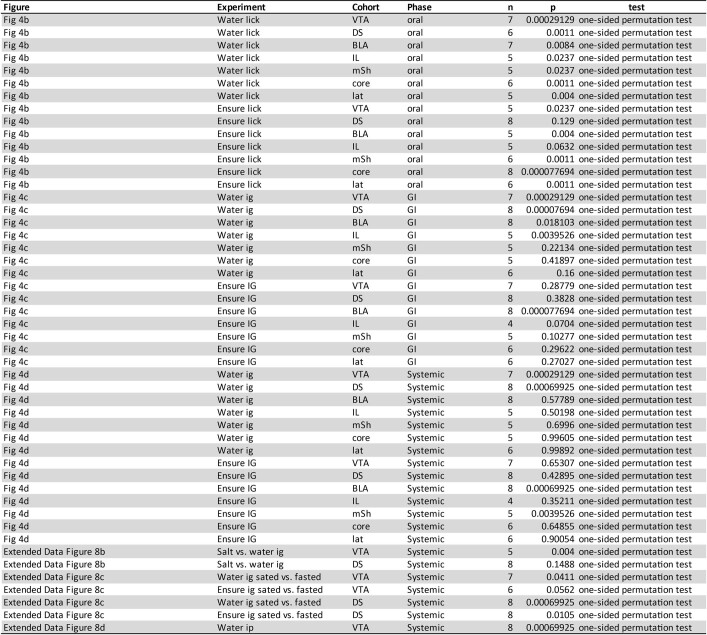
Statistics for photometry data

Statistics are shown for each variable quantified during a given phase of an experiment in each figure. The number of mice (n) recorded in the experimental cohort are given alongside the p-value measured by the given statistical test. Cohorts of mice are separated by the brain area in which GRAB-DA signals were being recorded. The phase is the time window during which activity was quantified. The “oral” time window was defined as the 30 s immediately following the first licking bout. The “GI” time window was defined asthe 12 min immediately following the onset of IG infusion. The “Systemic” time window was defined as the time over which the infused, or injected solution would be absorbed into the blood (12–50 min after the onset of IG infusion or self-paced consumption, 0–30 min after intraperitoneal injection). Note that exact permutation tests were used, resulting in no arbitrary limitation on lowest p-value obtainable. One-sided permutation tests were used in Figs. 4b–d to determine which areas had increased dopamine release in each experiment. One-sided permutation tests were used in Extended Data Figs. 8b–d where effect direction could be predicted based on VTA-DA neuron recordings. For example, since VTA-DA neuron activation after water IG infusion was greater if the mouse was dehydrated beforehand (Extended Data Fig. 1q), dopamine release in VTA was expected to follow the same trend. No corrections were made for multiple comparisons.

**Extended Data Table 4:**
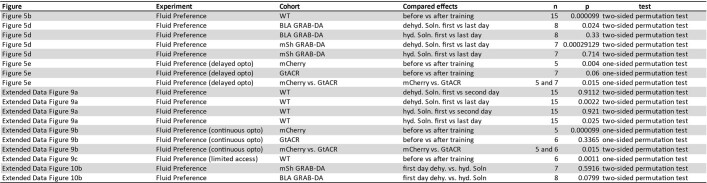
Statistics for fluid preference data

Statistics are shown for compared effects in each experiment in each figure using a given statistical test.The number of mice (n) recorded in the experimental cohort are given alongside the measured p-value. Note that exact permutation tests were used due to the relatively low n, resulting in no arbitrary limitation on lowest p-value obtainable. Two-sided permutation tests were initially used for fluid preference tests as no effect direction could be predicted. When particular effect directions were anticipated (as in subsequent fluid preference tests), one-sided permutation tests were used. No corrections were made for multiple comparisons.

## Data Availability

The data from this study are available from the corresponding author on reasonable request.

## Code availability

Links to the code used for data analysis are provided in the methods.
