# Supplementary material for: Dopamine subsystems that track internal states
Source: Nature. 2022 Jul 13;608(7922):374–80. doi: 10.1038/s41586-022-04954-0 (PMC9365689; doi:10.1038/s41586-022-04954-0)
Supplement: Supplementary file 1 — Reporting Summary [file 41586_2022_4954_MOESM1_ESM.pdf]

nature portfolio

Corresponding author(s): Zachary Knight

Last updated by author(s): Apr 29, 2022

Reporting Summary

Nature Portfolio wishes to improve the reproducibility of the work that we publish. This form provides structure for consistency and transparency in reporting. For further information on Nature Portfolio policies, see our Editorial Policies and the Editorial Policy Checklist.

Statistics

For all statistical analyses, confirm that the following items are present in the figure legend, table legend, main text, or Methods section.

n/a	Confirmed	
☐	☒	The exact sample size (*n*) for each experimental group/condition, given as a discrete number and unit of measurement
☐	☒	A statement on whether measurements were taken from distinct samples or whether the same sample was measured repeatedly
☐	☒	The statistical test(s) used AND whether they are one- or two-sided<br>*Only common tests should be described solely by name; describe more complex techniques in the Methods section.*
☐	☒	A description of all covariates tested
☐	☒	A description of any assumptions or corrections, such as tests of normality and adjustment for multiple comparisons
☐	☒	A full description of the statistical parameters including central tendency (e.g. means) or other basic estimates (e.g. regression coefficient) AND variation (e.g. standard deviation) or associated estimates of uncertainty (e.g. confidence intervals)
☐	☒	For null hypothesis testing, the test statistic (e.g. *F*, *t*, *r*) with confidence intervals, effect sizes, degrees of freedom and *P* value noted<br>*Give P values as exact values whenever suitable.*
☒	☐	For Bayesian analysis, information on the choice of priors and Markov chain Monte Carlo settings
☒	☐	For hierarchical and complex designs, identification of the appropriate level for tests and full reporting of outcomes
☒	☐	Estimates of effect sizes (e.g. Cohen's *d*, Pearson's *r*), indicating how they were calculated

Our web collection on statistics for biologists contains articles on many of the points above.

Software and code

Policy information about availability of computer code

Data collection	Synapse (TDT), Inscopix Data Acquisition Software (v151), Coulbourn Graphic State (v4.2)
Data analysis	Inscopix Data-Processing Software (v1.3.1), MathWorks Matlab (vR2020b), CNMF-E pipeline (http://www.github.com/zhoupc/cnmfe)

For manuscripts utilizing custom algorithms or software that are central to the research but not yet described in published literature, software must be made available to editors and reviewers. We strongly encourage code deposition in a community repository (e.g. GitHub). See the Nature Portfolio guidelines for submitting code & software for further information.

Data

Policy information about availability of data

All manuscripts must include a data availability statement. This statement should provide the following information, where applicable:

- Accession codes, unique identifiers, or web links for publicly available datasets
- A description of any restrictions on data availability
- For clinical datasets or third party data, please ensure that the statement adheres to our policy

Data that support the findings of this study are available from the corresponding author upon reasonable request.

Field-specific reporting

Please select the one below that is the best fit for your research. If you are not sure, read the appropriate sections before making your selection.

☒ Life sciences ☐ Behavioural & social sciences ☐ Ecological, evolutionary & environmental sciences

For a reference copy of the document with all sections, see nature.com/documents/nr-reporting-summary-flat.pdf

Life sciences study design

All studies must disclose on these points even when the disclosure is negative.

Sample size	Power calculations were used to predetermine sample size when prediction of effect size was possible based on previous experiments. Otherwise, we based sample sizes on previous studies from our lab and others.
Data exclusions	No data were excluded except in the following experiments. For plasma osmolality measurements, samples were thrown out if collection took over 2 minutes or if plasma supernatant had a lightness under 70% (see the color #ff6666 for reference). For inscopix recordings, videos of cell activity were removed if excessive motion occurred (larger than the diameter of the average neuron in any direction).
Replication	We confirmed our main findings by repeating recordings and behavioral experiments in multiple animals, and repeating all recordings in the same animal whenever possible. All attempts at replication were successful.
Randomization	Recordings were not randomized, but within-animal controls were used whenever possible. For the fluid preference tests, mice were randomly placed into four groups (2 factors x 2 levels), defined by order of solutions presented (factor 1) and which solution was paired with intragastric water infusion (factor 2). For VTA-DA silencing experiments, mice within each cage/litter were alternatively placed into experimental (GtACR) and control (mCherry) groups.
Blinding	The investigators were only blinded to allocation during VTA-DA silencing experiments Data analysis was performed automatically using the same Matlab scripts for each experimental group

Reporting for specific materials, systems and methods

We require information from authors about some types of materials, experimental systems and methods used in many studies. Here, indicate whether each material, system or method listed is relevant to your study. If you are not sure if a list item applies to your research, read the appropriate section before selecting a response.

Materials & experimental systems

n/a	Involved in the study	
☒	☐	Antibodies
☒	☐	Eukaryotic cell lines
☒	☐	Palaeontology and archaeology
☐	☒	Animals and other organisms
☒	☐	Human research participants
☒	☐	Clinical data
☒	☐	Dual use research of concern

Methods

n/a	Involved in the study	
☒	☐	ChIP-seq
☒	☐	Flow cytometry
☒	☐	MRI-based neuroimaging

Animals and other organisms

Policy information about studies involving animals; ARRIVE guidelines recommended for reporting animal research

Laboratory animals	We obtained DAT-ires-cre (B6.SJL-Slc6a3tm1.1(cre)Bkmn, Jackson #006660), Vgat-ires-cre (Slc32a1tm2(cre)Lowl, Jackson #016962), and wild type (Jackson #000664) mice from Jackson Labs. Vgat-ires-flp knock-in mice (B6.Cg-Slc32a1tm2.1(flop)Hze) and Npy-ires-flp knock-in mice (B6.Cg-Npytm1.1(flop)Hze) from the Allen Institute for Brain Science. DAT-ires-cre knock-in mice were crossed with Vgat-ires-flp knock-in mice or with Npy-ires-flp knock-in mice to generate double mutants.
Wild animals	No wild animals were used.
Field-collected samples	No field-collected samples were used.
Ethics oversight	Experimental protocols were approved by the University of California, San Francisco IACUC following the NIH Guide for the Care and Use of Laboratory Animals.

Note that full information on the approval of the study protocol must also be provided in the manuscript.

March 2021
